# Drug Errors and Protocol for Prevention among Anaesthetists in Nigeria

**DOI:** 10.1155/2017/2045382

**Published:** 2017-10-23

**Authors:** U. U. Johnson, L. N. Ebirim

**Affiliations:** Department of Anaesthesiology, University of Port Harcourt Teaching Hospital, PMB 6173, Port Harcourt, Rivers State, Nigeria

## Abstract

**Background:**

Drugs are often prescribed, dispensed, and administered by the same person during anaesthesia, and this may increase the risk of drug error.

**Objectives:**

To assess the frequency of drug administration errors by anaesthetists, the drugs commonly involved, and the effects of such errors.

**Method:**

A questionnaire-based study was carried out among participants at an annual conference of Nigerian anaesthetists. Sixty-six of the 80 participants returned the completed questionnaire. The respondents comprised 1 nurse anaesthetist, 34 resident doctors, 3 doctors with diploma in anaesthesia, and 28 consultant anaesthetists. The collated data on drug errors, the effect of such errors on patients, and formulated protocols to prevent future occurrence were subjected to descriptive analysis using Microsoft Excel.

**Result:**

Drug error was reported by 71.21% and witnessed by 22.72% of the respondents. Most of the drug errors occurred during general anaesthesia (90.3%) for emergency procedures (51.61%), and muscle relaxants were most commonly involved (58.06%).

**Conclusion:**

Drug errors are common among anaesthetists in Nigeria and their incidence is greater during general anaesthesia for emergency procedures, largely as a result of ampoule swaps due to similarities in ampoule design and packaging. Guidelines on their prevention should be developed by all health institutions.

## 1. Introduction

Anaesthesia is a unique specialty in which drugs are prescribed, dispensed, and often urgently administered by the same person, and this may increase the risk of some drugs being administered in error [1]. Morbidity and mortality which result from medication errors impact negatively on the confidence the populace has on the health care facility [2] and litigation can be instituted for claims and even imprisonment on grounds of negligence [3]. One of the solutions by World Health Organization for improving patients' safety involves reducing such errors. Unfortunately drug errors are often underreported [4], and plans to prevent their occurrence are not formulated. This study therefore sought to highlight the frequency, cause, and effect of drug administration errors among anaesthetists in Nigeria.

## 2. Method

A cross-sectional questionnaire-based study was carried out among participants at an annual scientific conference of anaesthetists in Nigeria. A copy of the questionnaire on drug errors in anaesthesia was given to each of 80 participants at the conference to complete. Only 66 completed copies of the questionnaire were returned. Data was collected on anaesthetic drugs administration errors, cause of the errors, effect on the patients, and formulated protocols to prevent future occurrence. The data was subjected to descriptive analysis using Microsoft Excel.

## 3. Result 

**Figure 1:**
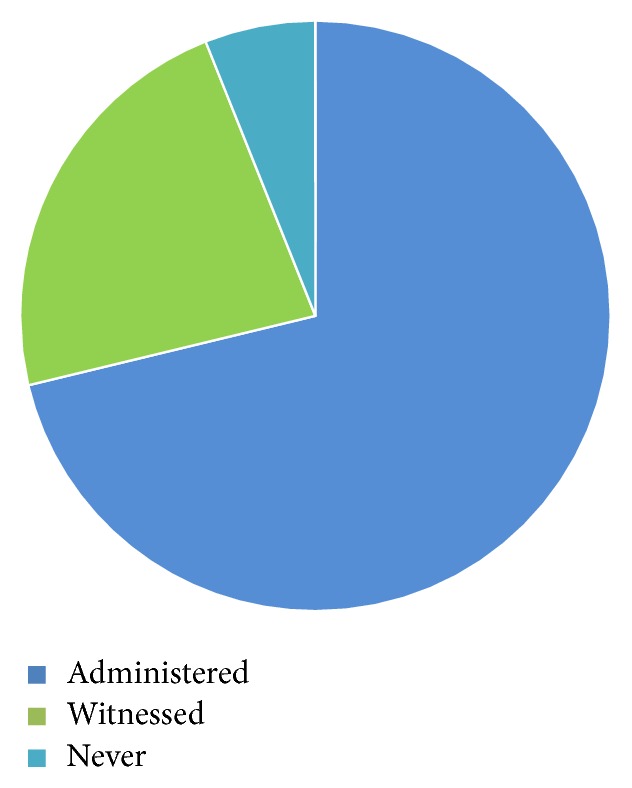
Incidence of drug errors.

Sixty-six (82.5%) of the 80 distributed copies of the questionnaire were completed and returned, and the respondents comprised 1 (1.51%) nurse anaesthetist, 34 (51.15%) resident doctors, 3 (4.54%) doctors with postgraduate diploma in anaesthesia, and 28 (42.42%) consultants with fellowship qualifications in anaesthesia. Forty-seven (71.21%) of the respondents had administered drug in error while 15 (22.72%) only witnessed and 4 (6.06%) neither administered nor witnessed drugs administered in error as shown in Figure 1.

Thirty-two (51.61%) of the errors occurred during emergency procedures, while 24 (38.70%) occurred during electives and 6 (9.67%) did not indicate the type of procedure. Fifty-six (90.32%) of the drug errors occurred during general anaesthesia, and 5 (8.06%) occurred during regional anaesthesia, while the type of anaesthesia was not indicated in 1 (1.61%). The drugs that were administered in error included premedicants, intravenous induction agents, muscle relaxants, analgesics, and vasopressors. Others included calcium injections and drugs for reversal of neuromuscular blockade as shown in Table 1.

Ampoules swaps accounted for 41 (66.12%) of the drug errors while syringe swaps were responsible for 15 (24.19%) and inappropriate dosing was recorded in 2 (3%) of the drug errors. An assistant was present in 45 (72.58%) of the cases, absent in 10 (16.12%), and not indicated in 7 (11.29%).

Effects of the drug errors were considered by the respondents to be mild in 16 (25.80%) cases, moderate in 22 (35.48%), severe in 9 (14.51%), and fatal in 2 (3.22%). In 13 (20.96%) of the cases there was no effect as shown in Table 2.

Guidelines for the prevention of drug error were not present in 8 (12.90%), but 54 (87.08%) used various methods either singly or combined as shown in Table 3.

## 4. Discussion 

Drug errors are considered to be uncommon but this may be an underestimation as a result of underreporting of incidents in the health care system [4], However, the estimated frequency of drug errors has been reported to be 1 in every 133 anaesthetics [5]. Most anaesthetists admit making at least 1 drug error in the course of their practice [1].

The drug errors could be inappropriate dosing, wrong sequence of administration, administration of a drug different from what was intended, or administration of a drug which the patient is allergic to. Drug errors could occur as a result of syringe swaps or ampoule swaps. One study [6] observed more syringe swaps (44%) than ampoule swaps (14%), and this was attributed to similarity in syringe colour and size but in this study the incidence of ampoule swaps was 69% while that of syringe swaps was 27%. Several studies have attributed ampoule swaps to similarities in drug packaging and ampoule design [7–10].

Drug error has been reported to be commoner during general anaesthesia than during regional anaesthesia [6]. In this study, 90.32% of the drug errors occurred during general anaesthesia while only 8.06% took place during regional/local anaesthesia. This is probably due to the fact that fewer drugs are used for regional than for general anaesthesia. There is a correlation between the urgency in drug administration and the incidence of drug error [11]. This explains the higher incidence of drug error observed during emergency procedures (51.61%, electives 24%) in this study.

It is important for the practitioner to develop an awareness of the ubiquity of medication errors and the potential capacity of such errors to inflict serious harm or death on patients [12]. The public perceives drug errors as negligence and suspension of the physician is considered an effective deterrent [2], and conviction for manslaughter could even be sought [13]. Education of staff is therefore crucial in the prevention of medication errors [14].

Understanding the causes and conditions that may lead to a medication error can help the practitioners to formulate a plan to prevent its occurrence. The causes of medication errors include lack of concentration by the attending physician, poor cart organization, ampoules looking alike, and unclear or unreadable drug labels [13]. Awareness should be created by the pharmacy department when drugs with similar label and packaging are procured to reduce the incidence of ampoule swaps. The legibility of some of the labels can be improved using magnifying glass and it was the practice by only 1.61% of the respondents. The use of magnifying glass can reduce the frequency of these errors and should therefore be encouraged.

Two-person confirmation or double check of drugs has been strongly advocated [15] and this was observed to be the practice in 46.77% of respondents in this study. However, double check can only be effective if the labels on the drugs are legible or readable and procurement of drugs with legible labels should be encouraged.

Another recommended method for confirming drugs before administration is the use of barcode technology [16]. Though very sensitive, the barcode technology is very expensive and cannot be afforded in the resource poor settings.

Medication errors can also be reduced by using syringe coding. Although it has been found [6] that syringe swaps occurred most often between syringes of equal size and the frequency was not reduced by introduction of colour coding of labels, standardized syringe sizes, with or without needles for particular drugs, can reduce the frequency of syringe swaps. Whereas the use of class-specific colour coding for syringe and ampoule might not reduce substitution of drugs in the same pharmacological class, it would have considerable potential for reducing interclass drug administration errors [17, 18]. About 6.45% of respondents in this study used only syringe coding. Various combinations of methods were used to prevent drug administration errors as indicated in Table 3.

Drug error has the potential for serious morbidity and even mortality. Whereas 20.96% were not affected by the drug errors reported, mild, moderate, and severe symptoms were observed in 25.80%, 35.48%, and 14.51%, respectively. Mortality was also reported in 3.22%. The death of any patient as a result of medication errors could be viewed as negligence by the public, and legal measures may be instituted against such personnel.

Despite many recommendations which have been made to minimize drug errors, their uptake in clinical practice is low. One barrier which has been consistently raised is that there remains a lack of class 1 evidence in favour of any of the recommended measures to reduce medication errors. Waiting for the evidence to emerge before implementing of change would inevitably run the risk of continuing significant patient harm [1]. However, a systematic review of the literature on the reduction of intravenous drug administration errors in anaesthesia has provided a strong support for the use of systematic countermeasures to decrease the incidence of intravenous drug administration errors in anaesthesia. Measures for the prevention of drug errors is therefore necessary and should be encouraged in our health institutions.

## 5. Conclusion

Drug administration errors are common among anaesthetists in Nigeria, and their incidence is greater during general anaesthesia for emergency procedures, largely as a result of ampoule swaps due to similarities in ampoule design and packaging. Legibility of the drug labels also contributed a significant percentage of drug errors. The pharmaceutical companies should therefore be encouraged to improve the ampoule design and legibility of labeling. Guidelines on the prevention of drug errors should be developed in all health institutions, and the use of magnifying lens for identification of drugs which was observed to be an uncommon practice in this environment should be encouraged.

## Figures and Tables

**Table 1:** Drug administered, type of error, and frequency.

Drugs administered in error	Type and frequency of error					
	Inappropriate dosing	Ampoules swaps	Syringe swaps	Not indicated	Total	Percentage
Premedicants	—	7	1	—	8	12.90
Induction agents	—	—	2	—	2	3.22
Muscle relaxants	—	25	11	—	36	58.06
Analgesics	1	—	—	—	1	1.61
Vasopressors	—	9	—	—	9	14.51
Calcium injection	1	—	—	—	1	1.61
Reversal drugs	—	—	1	—	1	1.61
Not Indicated	—	—	—	4	4	6.45

Total	2	41	15	4	62	100

**Table 2:** Effect of the drug errors.

Effect	Number of patients	Percentage
None	13	20.96
Mild	16	25.80
Moderate	22	35.48
Severe	9	14.51
Fatal	2	3.22

Total	62	100

**Table 3:** Preventive measures in respondents' health institutions.

Guidelines for prevention	Number of respondents	Percentage
None	8	12.90
All drugs must be administered by anaesthetist	1	1.61
Legible drug labels + double check	9	14.51
Syringe coding + double check	5	8.06
Double check only	29	46.77
Syringe coding + double checks + prefilled syringes with label	1	1.61
Syringe coding only	4	6.45
Legible drug labels only	1	1.61
Magnifying glass + double check	1	1.61
Magnifying glass + syringe coding	1	1.61
Magnifying glass + legible drug labels + double check	1	1.61
All the options	1	1.61

Total	62	100
